# Constrained Parameter Estimation for a Mechanistic Kinetic Model of Cobalt–Hydrogen Electrochemical Competition during a Cobalt Removal Process

**DOI:** 10.3390/e23040387

**Published:** 2021-03-24

**Authors:** Yiting Liang, Yuanhua Zhang, Yonggang Li

**Affiliations:** School of Automation, Central South University, Changsha 410083, China; liang_yiting@csu.edu.cn (Y.L.); 184612192@csu.edu.cn (Y.Z.)

**Keywords:** cobalt removal process, mechanistic kinetic model, parameter estimation, constrained parameter estimation

## Abstract

A mechanistic kinetic model of cobalt–hydrogen electrochemical competition for the cobalt removal process in zinc hydrometallurgical was proposed. In addition, to overcome the parameter estimation difficulties arising from the model nonlinearities and the lack of information on the possible value ranges of parameters to be estimated, a constrained guided parameter estimation scheme was derived based on model equations and experimental data. The proposed model and the parameter estimation scheme have two advantages: (i) The model reflected for the first time the mechanism of the electrochemical competition between cobalt and hydrogen ions in the process of cobalt removal in zinc hydrometallurgy; (ii) The proposed constrained parameter estimation scheme did not depend on the information of the possible value ranges of parameters to be estimated; (iii) the constraint conditions provided in that scheme directly linked the experimental phenomenon metrics to the model parameters thereby providing deeper insights into the model parameters for model users. Numerical experiments showed that the proposed constrained parameter estimation algorithm significantly improved the estimation efficiency. Meanwhile, the proposed cobalt–hydrogen electrochemical competition model allowed for accurate simulation of the impact of hydrogen ions on cobalt removal rate as well as simulation of the trend of hydrogen ion concentration, which would be helpful for the actual cobalt removal process in zinc hydrometallurgy.

Current zinc production is mainly based on zinc pyrometallurgy and zinc hydrometallurgy with the latter accounting for more than 80% of zinc production capacity [1]. Zinc hydrometallurgy consists of four processes: calcination, leaching, purification, and electrolysis. In the purification process, zinc dust is added for displacement deposition of impurity elements such as cobalt, nickel, and copper in the leaching solution thereby preventing co-deposition of impurity ions with zinc during the electrolysis process and the consequent decrease in electrolysis efficiency. The purification process involves the removal of copper, cobalt, and cadmium ions; cobalt ions are the most difficult to remove thereby making the cobalt removal step the most crucial.

The main reaction in the cobalt removal step is generally the electrochemical reaction of zinc–copper microcells [2,3,4]. Exposed zinc on the microcell surface undergoes oxidation to become zinc ions while cobalt ions obtain electrons and are reduced to elemental cobalt accompanied by reduction of hydrogen ions to hydrogen molecules. Low pH conditions (pH = 3–5) are usually adopted for cobalt removal to avoid zinc ions from forming basic zinc sulfate, which would deactivate the zinc dust by absorbing it onto its surface [5]. However, hydrogen ions have greater reactivity than cobalt ions on the zinc surface thereby making high-concentration hydrogen ions compete with cobalt ions for electrons result in a significant drop in the removal rate of cobalt [6]. To circumvent this competition, it is necessary to add copper salts, antimony salts, or arsenic salts as a catalyst to reduce the competitive reduction of hydrogen ions.

Despite such catalyst application, zinc dust in an amount twice the theoretically required amount should be used to decrease the concentration of cobalt ions to a proper level. The excess consumption of zinc dust arises from the competitive reduction of hydrogen ions. Given the large consumption of zinc dust in the purification process, how to model and estimate the cobalt removal process to achieve model-based optimal control of the cobalt removal process is an important research topic [7,8,9,10].

To model cobalt removal in the purification process of zinc hydrometallurgy, Wang [7] developed a first-order reaction kinetic model of cobalt removal assuming that the cobalt removal rate was proportional to the surface area of the zinc dust. They further used this model to develop a cobalt removal process model for continuous stirred-tank reactors (CSTRs) in series. Sun et al. [8] used the electrochemical mechanism of microcells in the cobalt removal process and treated the cathodic and anodic reactions in the cobalt removal process as independent processes under mixed potential control. They proposed a first-order electrochemical kinetic model based on mixed potential and seed concentration for the first time. Sun [9] proposed a modeling framework for strongly nonlinear processes based on multi-mechanism model synthesis, which nicely simulated the removal process of cobalt by arsenic salts. Li et al. [10] modeled cobalt removal via several interacting CSTR (ICSTR) systems with multiple time delays based on a mechanism of sharing the surface area of zinc dust in view of the competitive reduction of multiple ions in the cobalt removal process as well as the use of multiple stirred reactors in series. However, there remain multiple reactions in the zinc sulfate solution during the cobalt removal process: (1) competitive reduction of hydrogen ions and cobalt ions on the microcell surface; and (2) zinc ion hydrolysis that compensates for the loss of hydrogen ions due to reductive hydrogen evolution thereby slowing the rise of pH and affecting the cobalt removal rate. However, the cobalt removal process models proposed by Wang [7] and Sun [8,9] did not consider the competitive inhibitory effect of hydrogen ions on cobalt displacement, and the competitive reaction model was based on a mechanism of sharing the surface area of zinc dust [10]; it ignored the electrochemical nature of the competitive reaction. In addition, the impact of zinc hydrolysis on the concentration of hydrogen ions was ignored when modeling hydrogen reaction rates. These shortcomings are unfavorable for accurate assessment of the impact of pH fluctuation on zinc dust consumption and cobalt ion concentration during the cobalt removal process thereby adversely affecting optimal control of the cobalt removal process. Therefore, it is necessary to develop a kinetic model that not only incorporates the underlying electrochemical mechanism of the cobalt–hydrogen competitive reaction in the cobalt removal process but also reveals the trend of hydrogen ion concentration.

In addition to the difficulty in laying out the model structure, parameter estimates for the cobalt removal kinetic model also face many other challenges. The electrochemical reaction of various ions in the cobalt removal process and the coupling of hydrogen evolution with zinc ion hydrolysis are highly nonlinear processes. Parameter estimates for such strongly nonlinear dynamic systems usually occur through two classes of approaches.

The first class of approach estimate directly identifies the dynamic equations based on the time series of full-state values rather than solving the dynamic system [11,12]. For example, the SINDy algorithm [11] is a sparse estimation algorithm for nonlinear systems and estimates the first-order or even higher-order derivatives of the state according to available data. It then substitutes the derivatives into the state equation to estimate parameters. This approach is advantageous in that it avoids the strong nonlinearity arising from the integration of the dynamic system, but the disadvantage is that it requires high-frequency sampling of full-state data. For systems whose measurement values are partially missing or whose states are not all measurable, the state distribution at each time point can be recursively estimated through filtering and expectation-maximization followed by model parameter estimation through a state recursion equation [12,13]. However, this algorithm ensures convergence only when the initial estimates of parameters are relatively accurate.

The second class of approach is to solve the dynamic system and estimate model parameters by minimizing the differences between predicted outputs and measurements, which are usually unconstrained or simple constraints, like the upper and lower bounds of the parameters to be estimated. Different optimization algorithms may be involved in this approach—namely gradient-based optimization algorithms such as a sequential quadratic programming algorithm) versus heuristic optimization algorithms. Chai et al. [14] proposed a parameter estimation scheme for nonlinear time-delay dynamic systems that adopted a variational method to derive a formula for rapid gradient calculation greatly improving parameter estimation efficiency. However, gradient-based parameter estimation algorithms can only find locally optimal parameters and are not suitable for estimating model parameters of strongly nonlinear dynamic systems. Therefore, heuristic optimization algorithms such as particle swarm optimization (PSO) algorithms [15], state transition algorithms [16], differential evolution algorithms [17] and bee colony algorithm [18] have been widely used in parameter estimation of nonlinear systems. For example, Zhang [19] used a PSO algorithm to estimate parameters for a non-linear model of copper removal in the purification process of zinc hydrometallurgy. Deng et al. [20] used a state transition algorithm to estimate model parameters for the electrolysis process of zinc hydrometallurgy. In addition to heuristic optimization algorithms, additional prior information was incorporated into the estimation framework to narrow the parameter search space thus improving estimation efficiency and accuracy. Liu et al. [21] constructed a parameter optimization framework for a model of microbial batch fermentation that incorporated continuous state constraints according to the process mechanism and adopted an improved differential evolution algorithm to solve the estimation optimization problem. The continuous state constraints provide additional prior information for model parameter estimation to improve the reliability of estimation results. Improved differential evolution algorithms are more likely than gradient-based optimization algorithms to find globally optimal solutions, but they still use constraint penalty functions, which leads to a low estimation efficiency.

High-frequency sampling of state data is difficult for the cobalt removal process. Meanwhile, the kinetic and thermodynamic parameters in chemical databases only apply to specific experimental conditions (e.g., temperature, solution composition, and reactive electrode material) and model structures, which makes it difficult to accurately estimate the value range of a given parameter using chemical databases. These limitations prevent the algorithms above from being directly used in estimating the parameters of a given mechanistic kinetic model of the cobalt removal process. In the case of an unknown range of parameter values, a wide range of parameter searching is required to improve the solution accuracy. However, the non-linear dynamic system is solved each time that the objective function is calculated making the wide-range search time-consuming and inefficient and thereby limiting the estimation accuracy.

To overcome the obstacles to kinetic model development and parameter estimation of the cobalt removal process, firstly, this study developed a mechanistic kinetic model of cobalt–hydrogen electrochemical competition during the cobalt removal process. This model was composed of three models for the cobalt removal process: an electrochemical rate model, a mixed potential model of zinc dust, cobalt ions, and hydrogen ions, and a hydrolysis rate model of zinc ions. These models were all constructed in accordance with the principles of microcells and electrochemical kinetics. Secondly, this study proposed a constrained estimation scheme for model parameters. Specifically, mechanistic equations were simplified according to experimental data, and were then used to obtain approximate estimation equations for experimental metrics. Next, value ranges of experimental metrics were estimated according to experimental data to impose constraints on parameter estimation and narrow the parameter search space followed by transforming such constrained parameter estimation problem to an easy-to-solve box-constrained optimization problem through quasi-linear mapping of the parameters to be estimated. That is, the estimation problem of a given parameter with a difficult-to-estimate value range was transformed to the estimation problem of an experimental metric whose value range would be easily estimated from available experimental data. Meanwhile, the constraint conditions provided a relational expression directly linking the experimental metrics to the model parameters thereby providing deeper insights into the model parameters for model users.

The proposed parameter estimation scheme has three advantages. First of all, compared with the conventional unconstrained identification algorithm, the proposed constrained parameter estimation scheme does not depend on the information about the possible range of the parameters to be estimated. It obtains the searching space of parameters by building constraints of parameters based on a reasonable approximation of the experimental metrics of which rough ranges are easy to be obtained from available experimental data. As a result, the time consumption for searching optimal parameter value is dramatically reduced. Secondly, compared to the identification algorithms based on high-frequency sampling data, such as SINDy or filtering-based parameter estimation methods, it requires only low-frequency sampling experimental data. Thirdly, the constraint conditions provided by the scheme directly link the experimental phenomenon metrics to the model parameters, which provide a sense of intuition about how to adjust the parameters while the phenomenon changes in actual processes for model users.

The rest of this paper is organized as follows. In Section 1, a mechanistic kinetic model of cobalt–hydrogen electrochemical competition in the cobalt removal process is developed. In Section 2, a state-space model for cobalt removal in batch-type reactors is established based on the kinetic model and the principle of conservation of matter followed by introducing constraints into the model to construct a constrained parameter estimation problem and then transforming the problem into an easy-to-solve box-constrained optimization problem. In Section 3, the accuracy and efficiency of the proposed constrained parameter estimation algorithm in estimating model parameters based on experimental data are verified versus a conventional algorithm.

## 1. Cobalt–Hydrogen Electrochemical Competition Model for the Cobalt Removal Process

### 1.1. Mechanism Analysis of Cobalt Removal Process

**Figure 1:**
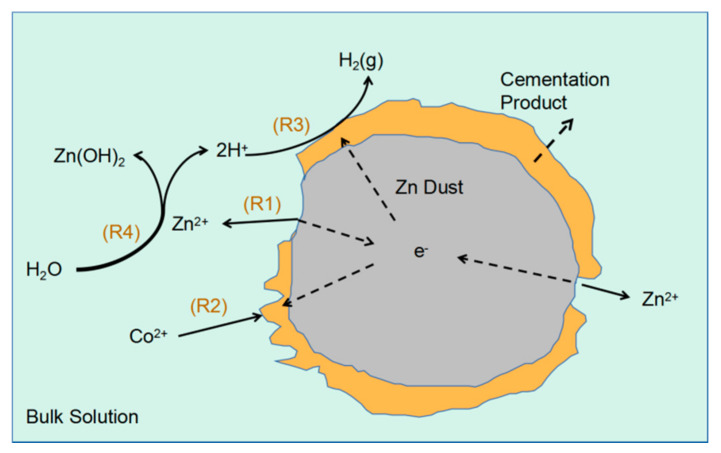
The micro-battery reaction mechanism of the cobalt removal process.

During zinc hydrometallurgy, the zinc ore is calcined and dissolved to form a zinc sulfate solution that is purified and sent for electrolysis to obtain pure zinc products. However, the zinc sulfate solution usually contains copper, cobalt, nickel, and other impurity ions. Of these, cobalt ions are the most difficult to remove, and excessive cobalt ions will cause a serious decrease in the efficiency of zinc electrolysis. Therefore, the cobalt removal step is a core step in the purification process of zinc metallurgy. The cobalt removal step is generally considered to involve electrochemical reactions on the surface of microcells as illustrated in Figure 1 [2,3,4]. Hydrogen ions have a high reactivity on the zinc and cobalt surfaces allowing hydrogen ions to compete with cobalt ions for electrons and thereby inhibit the displacement of cobalt. It is often necessary to add catalysts such as copper and antimony salts in the cobalt removal process to suppress the competitive hydrogen evolution on the zinc dust surface.

During the process of catalytic removal of cobalt by copper and antimony salts, the first reaction is the displacement of copper and antimony, which are deposited on the zinc dust surface to form active microcells. The displacement deposition of copper and antimony is very fast (complete within 5 min as reported by Lew [4]). Generally, the copper-antimony deposition is complete before cobalt begins to be deposited with cobalt deposition and hydrogen evolution on the microcells as well as zinc ion hydrolysis being the main reactions afterwards. The displacement of copper does not occur at the same time as that of copper and antimony; the competitive inhibitory effect of hydrogen ions on cobalt removal was the main focus of this study—we only investigated the electrochemical reactions of cobalt and hydrogen ions as well as zinc ion hydrolysis generating hydrogen ions. Three reversible half-cell reactions occur on the zinc-based microcell (Figure 1):

Zn^2+^ + 2e^−^↔Zn (1)

Co^2+^ + 2e^−^↔Co (2)

2H^+^ + 2e^−^↔H_2_ (3)

Zinc ion hydrolysis occurs in the main solution as shown by the following formula:

Zn^2+^ + 2H_2_O↔Zn(OH)_2_ + 2H^+^ (4)

In the actual process of cobalt removal, the net direction of half-cell Reaction (1) is to the left providing the electrons required for the evolution of hydrogen and displacement deposition of cobalt. Cobalt ions are displaced and deposited through a half-cell Reaction (2). Hydron evolution occurs through half-cell Reaction (3) in which hydrogen ions compete with cobalt ions for electrons reducing the cobalt removal rate. Zinc ion hydrolysis replenishes hydrogen ions as they are reduced and evolve as hydrogen gas exerting a buffering effect and indirectly affecting the cobalt removal rate.

### 1.2. Reaction Kinetics of Cobalt Removal

Almost all the data published [2,3,4,5,6] showed that the activation energy of cobalt removal reaction was higher than 40 kJ/mol, so it must be a chemically controlled process rather than a diffusion-controlled one. The electrochemical reaction for cobalt removal occurs on the microcell surface and is driven by the potential difference between the microcell surface and the solution. Given the very small diameters of the microcells and the high electrical conductivity of zinc and copper as the major microcell electrodes, we assumed that the potential at different areas on the microcell surface is identical, which is referred to as mixed potential and denoted as $E\left(t\right)$ in V. According to the principles of electrochemical kinetics—assuming that the total reactive surface area of microcells is proportional to the elemental zinc concentration—one can express the reverse rate of half-cell Reaction (1):

(5) $$r_{1}\left(c\left(t\right),E\left(t\right)\right)=\;k_{1}c_{\mathrm{Zn},0}\left(c_{{\mathrm{Zn}}^{2+}}\left(t\right)exp(-\frac{\left(2-\beta_{1}\right)FE\left(t\right)}{RT}\right)-b_{1}\frac{c_{\mathrm{Zn}}\left(t\right)}{c_{\mathrm{Zn},0}}exp(\frac{\beta_{1}FE\left(t\right)}{RT}))$$

where $c\left(t\right)$ represents the reactant concentration vector, $c\left(t\right)=\;{\left[c_{{\mathrm{Zn}}^{2+}}\left(t\right),c_{\mathrm{Zn}}\left(t\right),c_{{\mathrm{Co}}^{2+}}\left(t\right),c_{\mathrm{Co}}\left(t\right),c_{H^{+}}\left(t\right),c_{\mathrm{Zn}{\left(\mathrm{OH}\right)}_{2}}\left(t\right)\right]}^{T}$; $c_{M}\left(t\right)$, ${M\;=\;\mathrm{Zn}}^{2+}{,\;\mathrm{Zn},\;\mathrm{Co}}^{2+}{,\;\mathrm{Co},\;H}^{+},\;\mathrm{Zn}{\left(\mathrm{OH}\right)}_{2}$ is the concentration of reactant M in mol/L; $r_{i},i=1,\ldots ,4$ is the rate of reaction (i), which is a function of $c\left(t\right)$ and $E\left(t\right)$; $c_{\mathrm{Zn},0}$ is the initial concentration of elemental zinc in mol/L; $k_{1}$ is the rate constant of Reaction (1); $b_{1}$ is the equilibrium constant of Reaction (1); $\beta_{1}$ is the anode transfer coefficient of Reaction (1); F is the Faraday constant (96,485 C/mol); *R* is the gas constant (8.314); and *T* is the reaction temperature (346 K in this study).

Assuming that cobalt displacement is determined by the electrochemical reaction and that the number of electrons involved is 2, the reaction rate of half-cell Reaction (2) can be expressed as:

(6) $$r_{2}\left(c\left(t\right),E\left(t\right)\right)=\;k_{2}c_{\mathrm{Zn},0}\left(c_{{\mathrm{Co}}^{2+}}\left(t\right)exp(-\frac{\alpha_{2}E\left(t\right)F}{RT}\right)-b_{2}\frac{c_{\mathrm{Co}}\left(t\right)}{c_{\mathrm{Zn},0}}exp(\frac{\left(2-\alpha_{2}\right)E\left(t\right)F}{RT}))$$

where $k_{2}$ is the rate constant of Reaction (2), $b_{2}$ is the equilibrium constant of Reaction (2), and $\alpha_{2}$ is the cathode transfer coefficient of Reaction (2).

Given that the reverse of hydrogen evolution Reaction (3) is usually weak and can be ignored, only the forward reaction is considered in this study. Accordingly, the rate of hydrogen evolution reaction is:

(7) $$r_{3}\left(c\left(t\right),E\left(t\right)\right)=\;k_{3}c_{\mathrm{Zn},0}c_{H^{+}}\left(t\right)exp(-\frac{\alpha_{3}FE\left(t\right)}{RT})$$

where $k_{3}$ is the rate constant of Reaction (3), $b_{3}$ is the equilibrium constant of Reaction (3), and $\alpha_{3}$ is the cathode transfer coefficient of Reaction (3).

According to the principle of an electric double layer (EDL), the rate of change of mixed potential of a microcell is proportional to the total electrochemical current on the microcell as follows:

(8) $$\dot{E}\left(t\right)=\;C^{-1}F\overset{3}{\underset{i=1}{\sum }}2r_{i}\left(c\left(t\right),E\left(t\right)\right)$$

where C is the capacitance of the EDL. The rate of hydrolysis Reaction (4) of zinc ions is:

(9) $$r_{4}\left(c\left(t\right),E\left(t\right)\right)=\;k_{4}(c_{{\mathrm{Zn}}^{2+}}\left(t\right)-b_{4}c_{\mathrm{Zn}{\left(\mathrm{OH}\right)}_{2}}\left(t\right)c_{H^{+}}\left(t{)}^{2}\right)$$

where $k_{4}$ is the rate constant of Reaction (4), and $b_{4}$ is the equilibrium constant of Reaction (4).

Formulas (5)–(9) constitute a kinetic model of cobalt–hydrogen electrochemical competition in the cobalt removal process, which contain many unknown parameters and whose estimation algorithms will be elaborated in the next section.

## 2. Constrained Parameter Estimation Algorithms for The Cobalt–Hydrogen Electrochemical Competition Model

For the cobalt–hydrogen electrochemical competition model, the value of hydrolysis rate constant $k_{4}$ was set to $1\times 10^{8}$ in this study considering that its fluctuation within a certain range would have little impact on modeled concentration of cobalt and hydrogen ions. In addition, the charge and discharge time of a microcell was assumed to be less than the reaction characteristic time, i.e., $FC^{-1}$ was relatively large. In such a scenario, the fluctuation of $FC^{-1}$ within a certain range would have little impact on model results and accordingly its value was set to 1 × 10^4^. The important parameter vector to be estimated to be:

(10) $$p^{\left(0\right)}\;=\;\left[\beta_{1},\alpha_{2},\alpha_{3},k_{1},k_{2},k_{3},b_{1},b_{2},b_{4}\right]$$

In view of the great order of magnitudes in the fluctuation of reaction rate constants and equilibrium constants, the last six constants in $p^{\left(0\right)}$ were logarithm-transformed thereby leading to the following estimates of the parameter vector:

(11) $$p^{\left(1\right)}\;=\;\left[\beta_{1},\alpha_{2},\alpha_{3},ln(k_{1}\right),ln(k_{2}),ln(k_{3}),ln(b_{1}),ln(b_{2}),ln(b_{4})]$$

Lew and colleagues [4] performed experimental tests under 20 conditions to explore the impacts of copper and antimony contents, pH, zinc dust dosage, and zinc dust particle size on the efficiency of batch removal of cobalt under catalysis by copper and antimony. They obtained abundant kinetic data on the cobalt removal process. Experimental data under five conditions regarding the impact of pH on cobalt removal rate in that study were taken as source data to estimate the parameters of the present mechanistic kinetic model. These experimental data were the monitored values of cobalt ion concentration at constant pH values of 3.0, 3.6, 4.0, and 4.4 as well as the monitored values of cobalt ion concentration and pH under an uncontrolled pH condition (Page 62, Figure 4-31; Page 47, Figure 4-10; page 42, Figure 4-4). Since the cobalt removal tests by Lew [4] were performed in batch reactors, a state-space model will be developed in Section 2.1 based on the cobalt–hydrogen competition model developed in Section 1 to describe the batch removal process of cobalt. Section 2.2 will construct a parameter estimation problem based on the state-space model and address the difficulty in solving the problem. Section 2.3 proposes constraint conditions to make the above parameter estimation problem become a constrained parameter estimation problem. Section 2.4 further transforms the problem into an easy-to-solve box-constrained optimization problem.

### 2.1. State Space Model for the Batch Removal Process of Cobalt

For a given half-cell reaction, its state function is constructed in terms of the cumulative concentration change of the reactant as follows:

(12) $${\dot{x}}_{i}\left(t\right)\;=\;r_{i}\left(c\left(t\right),E\left(t\right)\right),i=1,\ldots ,4$$

where *t* represents time in min, and $x_{i}\left(t\right)$ the cumulative concentration changes of the reactant of reaction (*i*) in mol/L. The material balance equation in the batch reactor is:

(13) $$c\left(t\right)\;=\;c_{0}+\Lambda x\left(t\right)$$

where $c_{0}$ is the vector of the initial concentration of reactants added in batch:

$$c_{0}\;={\left[c_{{\mathrm{Zn}}^{2+},0},c_{\mathrm{Zn},0},c_{{\mathrm{Co}}^{2+},0},c_{\mathrm{Co},0},c_{H^{+},0},c_{\mathrm{Zn}{\left(\mathrm{OH}\right)}_{2},0}\right]}^{T}$$

Here, $c_{M,0}$ (M = Zn^2+^, Zn, Co^2+^, Co, H^+^, Zn(OH)_2_) is the initial concentration of reactant M in mol/L, and Λ is a stoichiometric coefficient matrix where column *i* represents the stoichiometric coefficients of all the six reactants for reaction (*i*), *i* = 1, 2, 3, and 4, based on Equations (1)–(4):

(14) $$\Lambda \;=\;\left[\begin{array}{cccc} -1 & 0 & 0 & -1\\ 1 & 0 & 0 & 0\\ 0 & -1 & 0 & 0\\ 0 & 1 & 0 & 0\\ 0 & 0 & -2 & 2\\ 0 & 0 & 0 & 1 \end{array}\right]$$

According to Equations (8) and (12), the mixed potential in the batch reactor is:

(15) $$E\left(t\right)=\;E_{0}+FC^{-1}\left[\begin{array}{ccc} 2 & 2 & 2 \end{array}\right]x\left(t\right)$$

where $E_{0}$ is the initial mixed potential in V. Considering that the experimental data used for model parameter estimation in this study included cobalt ion concentration and pH, the model output is:

(16) $$y\left(t\right)\;=\;\left[\begin{array}{c} c_{{\mathrm{Co}}^{2+}}\left(t\right)\\ c_{H^{+}}\left(t\right) \end{array}\right]\;=\;\left[\begin{array}{cccccc} 0 & 0 & 1 & 0 & 0 & 0\\ 0 & 0 & 0 & 0 & 1 & 0 \end{array}\right]c_{0}+\left[\begin{array}{cccc} 0 & -1 & 0 & 0\\ 0 & 0 & -2 & 2 \end{array}\right]x\left(t\right)$$

Equations (12)–(16) constitute a state-space model of the batch removal process of cobalt where the state vector is x and the output is y. For the convenience of description, function *G* is introduced to represent the relationship between the parameters, the initial concentration of reactants, and the model output in the state-space model (13) of the batch removal process of cobalt as follows:

(17) $$y\left(t\right)\;=\;G(t|p,c_{0})$$

Since the experimental data used for model parameter estimation in this study also included the experimental data of batch removal of cobalt under constant-pH conditions, it is necessary to develop a state-space model for such removal processes under constant-pH conditions. Accordingly, the hydrogen ion concentration is set to a constant value in the state space model Equation (13) followed by solving the equations to obtain the model output. Specifically, Equations (13) and (16) are replaced by Equations (18) and (19), respectively:

(18) $$c\left(t\right)\;=\;c_{0}+\Lambda^{*}x\left(t\right)$$

(19) $$y\left(t\right)\;=\;\left[\begin{array}{c} c_{{\mathrm{Co}}^{2+}}\left(t\right)\\ c_{H^{+}}\left(t\right) \end{array}\right]\;=\;\left[\begin{array}{cccccc} 0 & 0 & 1 & 0 & 0 & 0\\ 0 & 0 & 0 & 0 & 1 & 0 \end{array}\right]c_{0}+\left[\begin{array}{cccc} 0 & -1 & 0 & 0\\ 0 & 0 & 0 & 0 \end{array}\right]x$$

In Equation (18), one has:

(20) $$\Lambda^{*}\;=\;\left[\begin{array}{cccc} -1 & 0 & 0 & -1\\ 1 & 0 & 0 & 0\\ 0 & -1 & 0 & 0\\ 0 & 1 & 0 & 0\\ 0 & 0 & 0 & 0\\ 0 & 0 & 0 & 1 \end{array}\right]$$

To simplify subsequent expressions, the relationship between the parameters obtained based on Equations (12), (15), (18), (19) and (20), the initial concentration of the reactants, and the model output is expressed as:

(21) $$y\left(t\right)\;=\;G^{*}(t|p,c_{0})$$

### 2.2. Parameter Estimation Problem of Cobalt–Hydrogen Electrochemical Competition System

For electrochemical rate Equations (5)–(7), the cathode and anode reaction transfer coefficients are confined in the following ranges:

$$\beta_{1}\in \left[0.7,1.5\right],\alpha_{2}\in \left[0.2,0.8\right],\alpha_{3}\in \left[0.2,0.8\right]$$

**Figure 2:**
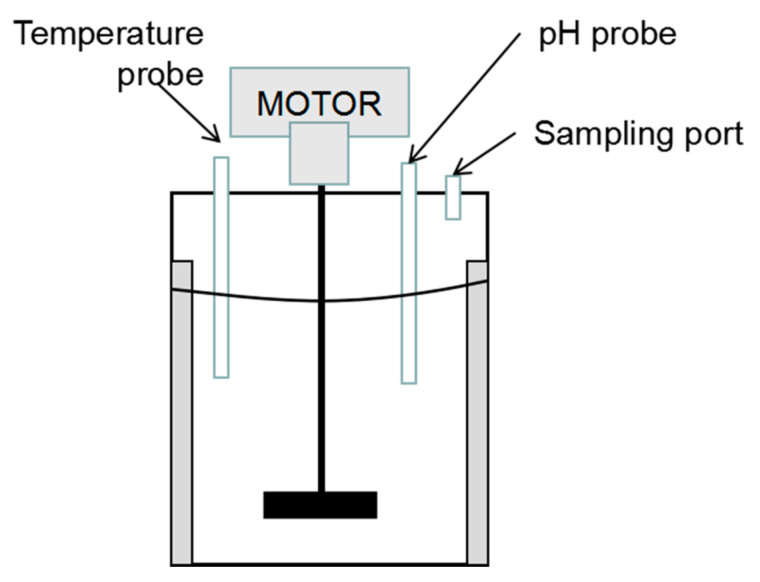
The batch reactor for cobalt removal experiments of Lew [4].

In order to identify the model parameters, experimental data published in Lew [4], a doctoral thesis on the mechanism of the copper-antimony activated cobalt removal from zinc sulphate solutions, were used in this paper. In Lew [4], the cobalt removal experiments were carried out in a batch stirred reactor, as shown in Figure 2. The volume of the reactor is 4 L, and the stirring speed is 700 RPM. The main component of the reaction solution was ZnSO_4_, and a certain amount of copper, antimony and cobalt ions were added one time in advance according to the design of each experiment. Zinc powder was added at one time at the initial stage of the reaction. During the experiments, the cobalt ion concentration was measured by sampling using a 50-mL pipette at intervals of 5, 15, 30, 45, 60 and 90 min, and the pH values are monitored online. The data from five experiments are selected from Lew [4] for model identification: experiment with natural pH (initial pH = 3.7), controlled pH (pH = 3.0), controlled pH (pH = 3.6), controlled pH (pH = 4.0), and controlled pH (pH = 4.4). In general, the experimental setup of the data used by this study was performed under the following conditions:

1. 4 g/L loading with −100 + 140 mesh (126 to 174 micron) zinc dust;
2. The batch reactor was heated in a thermostated water bath and maintained to within 1 °C of the target value of temperature 73 °C;
3. Natural pH: the pH was allowed to come to its own equilibrium from a starting pH of 3.7;
4. Controlled pH: the pH was controlled to be within 0.2 units of the target value using a pH controller;
5. 26 mg/L Co;
6. 46 mg/L Cu, 1.5 mg/L Sb were added as catalysts.

Accordingly, the following parameter estimation problem is proposed:

(22) $$\underset{p^{\left(1\right)}}{min}\overset{5}{\underset{j=1}{\sum }}\overset{n_{\mathrm{Co},j}}{\underset{i=1}{\sum }}\left(\frac{1}{n_{\mathrm{Co},j}}\left|ln(\frac{c_{{\mathrm{Co}}^{2+},0}^{\left(j\right)}}{{\hat{c}}_{{\mathrm{Co}}^{2+}}^{\left(j\right)}\left(t_{i,{\mathrm{Co}}^{2+}}^{\left(j\right)}\right)})-ln(\frac{c_{{\mathrm{Co}}^{2+},0}^{\left(j\right)}}{c_{{\mathrm{Co}}^{2+},i}^{\left(j\right)}})\right|\right)+\frac{\lambda_{\mathrm{pH}}}{n_{\mathrm{pH}}}\overset{n_{\mathrm{PH}}}{\underset{l=1}{\sum }}\left|-{log}_{10}({\hat{c}}_{H^{+}}^{\left(1\right)}\left(t_{l,H^{+}}^{\left(1\right)}\right))-{\mathrm{pH}}_{l}\right|\;s.t.\left[\begin{array}{c} {\hat{c}}_{{\mathrm{Co}}^{2+}}^{\left(j\right)}\left(t\right)\\ {\hat{c}}_{H^{+}}^{\left(j\right)}\left(t\right) \end{array}\right]\;=\;\left\{\begin{array}{c} G(t|p^{\left(1\right)},c_{0}^{\left(j\right)}),\mathrm{if}\;j=1,\\ G^{*}(t|p^{\left(1\right)},c_{0}^{\left(j\right)}),\mathrm{if}\;j>1, \end{array}\right.\;\beta_{1}\in \left[0.7,1.5\right],\alpha_{2}\;\in \;\left[0.2,0.8\right],\alpha_{3}\in \left[0.2,0.8\right].$$

where *j* = 1,2,3,4,5 represent natural pH, controlled pH (pH = 3.0), controlled pH (pH = 3.6), controlled pH (pH = 4.0), and controlled pH (pH = 4.4), respectively. For the natural pH condition, cobalt concentration data (Page 47, Figure 4-10) and pH values (page 42, Figure 4-4) were collected while for the other four controlled-pH conditions (*j* = 2,3,4,5) only cobalt concentration data were collected (Page 62, Figure 4-31). $c_{0}^{\left(j\right)}$ represents the initial concentration in test *j*; ${\hat{c}}_{{\mathrm{Co}}^{2+}}^{\left(j\right)}\left(t\right)$ and ${\hat{c}}_{H^{+}}^{\left(j\right)}\left(t\right)$ represent the model-predicted concentration of cobalt ion and hydrogen ion in test *j*, respectively; $c_{{\mathrm{Co}}^{2+},i}^{\left(j\right)},$ and pH*_l_* represent the *i*-th measurement value of cobalt ion concentration and the *l*-th measurement value of pH in test *j*, respectively; $t_{i,{\mathrm{Co}}^{2+}}^{\left(j\right)}$ and $t_{l,H^{+}}^{\left(j\right)}$ represent the measurement time of cobalt ion concentration and pH in test *j*, respectively; $\lambda_{\mathrm{pH}}$ is the weight coefficient of pH; $n_{\mathrm{pH}}$ is the number of collected pH values in test 1; and $n_{\mathrm{Co},j}$ is the number of collected concentration values of cobalt ions in test *j*.

The model parameters can be estimated by solving Equation (22), which is very difficult to solve. The nonlinearity of mechanistic kinetic Equations (5)–(9) makes Equation (22) a non-convex optimization problem, which is difficult to directly solve via gradient-based optimization algorithms (such as sequential quadratic programming algorithms and interior-point algorithms); however, it is usually solvable by heuristic optimization algorithms only when the ranges of parameter values are known. However, since the kinetic and thermodynamic parameters in the standard chemical databases apply to specific experimental conditions, such as temperature, solution composition, electrode matrix composition, and kinetic models, it is difficult to find corresponding values from chemical databases for the present model parameters (i.e., $k_{1},k_{2},k_{3},b_{1},b_{2},b_{4}$) thereby making it impossible to estimate the reasonable value ranges of the parameters. To ensure that the optimal parameters are found, it is necessary to give a large parameter search range, but a large range search will result in low optimization efficiency. A more serious issue is that there is coupling between the competitive electrochemical reactions, the hydrogen evolution, and the zinc ion hydrolysis in the cobalt–hydrogen electrochemical competition mechanism model, which forms a highly nonlinear process. As a result, there would be large differences in reaction characteristic time between reactions (1)–(4) when model parameters are randomly set to a value, thereby causing the state-space model of batch reactor to achieve a very stiff dynamic system. A trial solution of such a system is time-consuming and results in low optimization search efficiency.

The key to overcoming the limitations above is to accurately determine the parameter value ranges. Although it is impossible to directly obtain the value ranges of the model parameters, it is still possible to experimentally estimate a reasonable value range using some experimental data measured under certain conditions such as the mixed potential within a certain period of time, the apparent first-order reaction rate constant, the equilibrium concentration, and the change of the rate of solid deposition or gas evolution at a certain time. Provided that an approximate relationship between these experimental data and the model parameters is obtained, it would be possible to further impose constraints on the parameters to rapidly determine a reasonable range of parameter values and improve the solution efficiency.

In the next sub-section, assumptions are made based on experimental data to simplify the mechanistic equations so that they can be partially yet directly solved. The solutions are directly used to establish approximate expressions linking the model parameters to some experimental metrics. Next, constraints are introduced for the model parameters by specifying value ranges of the experimental metrics based on experimental data, thereby leading to a constrained parameter estimation scheme.

### 2.3. Constrained Parameter Estimation Algorithm

#### 2.3.1. Constraints on Electrochemical Parameters

In this sub-section, the cobalt removal experimental data at pH 3 reported by Lew [4] (Section 4, Page 60–62) will be analyzed in view of the test conditions so as to introduce proper consumptions for simplifying the reaction kinetic models. Next, the mixed potential, the apparent first-order reaction rate constant, and equilibrium cobalt concentration were estimated as experimental metrics according to the simplified kinetic equations to impose constraints on relevant electrochemical parameters of cobalt and hydrogen. The mixed-potential change rate Equation (8) is simplified. Based on the assumption that the charge and discharge of the EDL capacitor are fast (i.e., $FC^{-1}$ is sufficiently large), the charge and discharge time is ignored thereby allowing Equation (8) to be approximated by a balance equation:

(23) $$\overset{3}{\underset{i=1}{\sum }}r_{i}\left(c\left(t\right),E\left(t\right)\right)=\;0$$

As indicated by the above equation, the total number of electrons lost through oxidation on a microcell is equal to the total number of electrons obtained through reduction. According to Lew [4] (Page 61, Table 4-2), the first-order reaction rate constant of cobalt was about $489\times 10^{-6}\;\sec^{-1}$ pH 3. The first-order reaction rate constant of hydrogen was estimated here to be $767\times 10^{-5\;}\sec^{-1}$ based on the early-stage portion (0–5 min) of the hydrogen evolution curve reported by Lew [4] (Page 70, Figure 4-42) with zero initial concentration of zinc ions. This was about 15 times that of cobalt. In the test at pH 3, the hydrogen ion concentration was approximately 2.26-times the cobalt ion concentration (26 mg/L). Considering that two electrons are consumed to displace a single cobalt ion while one electron is required for the reduction of a single hydrogen ion, the current of hydrogen evolution Reaction (3) is approximately 15×2.26/2 that of cobalt reduction (2). The concentration of cobalt ions became smaller with the passage of time. However, the concentration of hydrogen ions remained nearly constant under constant-pH conditions, which makes the current of Reaction (3) increasingly large relative to that of Reaction (2). Consequently, the current of Reaction (2) would have little impact on the mixed potential, which was largely affected by Reaction (3) and zinc dissolution Reaction (1). That is, the coefficient $r_{2}$ in Equation (23) can be ignored. In addition, the reverse rate of zinc dissolution Reaction (1) is assumed to be ignorable when estimating the mixed potential thereby allowing Equation (23) to be further simplified:

(24) $$k_{1}c_{\mathrm{Zn},0}\left(-b_{1}\frac{c_{\mathrm{Zn}}}{c_{\mathrm{Zn},0}}exp(\frac{\beta_{1}FE}{RT}\right))+k_{3}c_{\mathrm{Zn},0}c_{H^{+}}exp(-\frac{\alpha_{3}FE}{RT})\;=\;0$$

The mixed potential can be estimated from Equation (24):

(25) $$E_{\mathrm{est}}\;=\;RTF^{-1}{\left(\beta_{1}+\alpha_{3}\right)}^{-1}\left(ln(k_{3}c_{\mathrm{Zn},0}c_{H^{+}})-ln(b_{1}k_{1}c_{\mathrm{Zn}})\right)$$

The experimental data of Lew [4] (Page 61, Figure 4-29) showed that the potentiometer reading was approximately −0.6 V for sludge at pH 3 ($c_{H^{+}}=10^{-3}\;\mathrm{mol}/L$); thus, we assumed that $E_{\mathrm{est}}\in \left[-0.7,-0.5\right]$. In addition, the amount of zinc dust added during the cobalt removal reaction is excessive (more than 20 times the amount of zinc dust theoretically required for displacement of copper, antimony, and cobalt ions). Thus, we assumed that the content of zinc dust is roughly constant, namely $c_{\mathrm{Zn}}\approx c_{\mathrm{Zn},0}$. Substitution of this expression in Equation (25) leads to the following constraint:

(26) $$-\left(\beta_{1}+\alpha_{3}{)}^{-1}\left(ln(b_{1}k_{1}k_{3}^{-1}\right)+3ln(10\right))\;\in \;[-0.7F{\left(RT\right)}^{-1},-0.5F\left(RT{)}^{-1}\right]$$

Considering that the reverse rate of the displacement reaction of cobalt ions at the early stage is negligible compared to the forward rate, the displacement reaction rate of cobalt ions can be estimated using mixed-potential estimation Equation (25) as follows:

(27) $$r_{2}\;\approx \;k_{2}c_{\mathrm{Zn},0}c_{{\mathrm{Co}}^{2+}}{\left(b_{1}k_{1}c_{\mathrm{Zn}}\right)}^{\frac{\alpha_{2}}{\beta_{1}+\alpha_{3}}}{\left(k_{3}c_{\mathrm{Zn},0}c_{H^{+}}\right)}^{-\frac{\alpha_{2}}{\beta_{1}+\alpha_{3}}}$$

Assuming that the content of zinc dust is roughly constant, namely $c_{\mathrm{Zn}}\approx c_{\mathrm{Zn},0}$, the apparent first-order reaction rate constant of cobalt displacement reaction can be calculated as follows:

(28) $${\tilde{k}}_{2}\;\approx \;k_{2}c_{\mathrm{Zn},0}{\left(b_{1}k_{1}\right)}^{\frac{\alpha_{2}}{\beta_{1}+\alpha_{3}}}{\left(k_{3}c_{H^{+}}\right)}^{-\frac{\alpha_{2}}{\beta_{1}+\alpha_{3}}}$$

The experimental data of Lew [4] (Page 61, Table 4-2) showed that at pH 3, the apparent first-order reaction rate constant of cobalt displacement reaction was 0.02934 min^−1^. Considering the model simplification error, one may let ${\tilde{k}}_{2}\in [0.015\;\min^{-1},0.060\;\min^{-1}]$. Based on Equation (28), the following constraint is obtained:

(29) $$\alpha_{2}\left(\beta_{1}+\alpha_{3}{)}^{-1}\left(ln(k_{1})+ln(b_{1})-ln(k_{3})+3ln(10)\right)+ln(k_{2}\right)+ln(c_{\mathrm{Zn},0})\;\in \;\left[ln(0.015\right),ln(0.060)]$$

The rate of hydrogen evolution reaction is:

(30) $$r_{3}\;\approx \;k_{3}c_{\mathrm{Zn},0}c_{H^{+}}{\left(b_{1}k_{1}c_{\mathrm{Zn}}\right)}^{\frac{\alpha_{3}}{\beta_{1}+\alpha_{3}}}{\left(k_{3}c_{\mathrm{Zn},0}c_{H^{+}}\right)}^{-\frac{\alpha_{3}}{\beta_{1}+\alpha_{3}}}$$

Accordingly, the apparent first-order rate constant of hydrogen evolution reaction is:

(31) $${\tilde{k}}_{3}\;\approx \;k_{3}c_{\mathrm{Zn},0}{\left(b_{1}k_{1}c_{\mathrm{Zn}}\right)}^{\frac{\alpha_{3}}{\beta_{1}+\alpha_{3}}}{\left(k_{3}c_{\mathrm{Zn},0}c_{H^{+}}\right)}^{-\frac{\alpha_{3}}{\beta_{1}+\alpha_{3}}}$$

As shown by the earlier analysis of the experimental data of Lew [4] in this sub-section, the rate constant of hydrogen evolution reaction was about 15 that of cobalt reduction at pH = 3. In view of the estimation error, the following constraint assumption is introduced for parameter estimation: At pH 3, the apparent pseudo-first-order rate of hydrogen evolution reaction is between 5–50 times the apparent first-order rate of cobalt reduction, namely ${\tilde{k}}_{3}{\left({\tilde{k}}_{2}\right)}^{-1}\in \left[5,50\right]$. Taking logarithms of both sides based on Equations (31) and (28) while assuming that $c_{\mathrm{Zn}}\approx c_{\mathrm{Zn},0}$ yields the following constraint:

(32) $$\left(\alpha_{3}-\alpha_{2}\right)\left(\beta_{1}+\alpha_{3}{)}^{-1}\left(ln(b_{1}k_{1}k_{3}^{-1})+3ln(10)\right)+ln(k_{3}k_{2}^{-1}\right)\;\in \;\left[ln(5\right),ln(10)]$$

Substituting mixed-potential estimation expression (25) into the reaction rate Equation (6) of cobalt displacement Reaction (2) and letting $r_{2}=0$, the equilibrium concentration of cobalt can be calculated as:

(33) $$c_{{\mathrm{Co}}^{2+},\mathrm{eq}}\;=\;\frac{b_{2}{\left(k_{3}c_{\mathrm{Zn},0}c_{H^{+}}\right)}^{\frac{2}{\beta_{1}+\alpha_{3}}}{\left(b_{1}k_{1}c_{\mathrm{Zn}}\right)}^{-\frac{2}{\beta_{1}+\alpha_{3}}}}{b_{2}{\left(k_{3}c_{\mathrm{Zn},0}c_{H^{+}}\right)}^{\frac{2}{\beta_{1}+\alpha_{3}}}{\left(b_{1}k_{1}c_{\mathrm{Zn}}\right)}^{-\frac{2}{\beta_{1}+\alpha_{3}}}+c_{\mathrm{Zn},0}}c_{{\mathrm{Co}}^{2+},0}$$

According to the experimental data of Lew [4] (Page 47, Figure 4-10), $c_{{\mathrm{Co}}^{2+},\mathrm{eq}}\leq 0.1c_{{\mathrm{Co}}^{2+},0}$. Accordingly, the first term in the denominator of the above equation can be ignored. Moreover, assuming $c_{\mathrm{Zn}}\approx c_{\mathrm{Zn},0}$:

(34) $$c_{{\mathrm{Co}}^{2+},\mathrm{eq}}\;\approx \;b_{2}{\left(k_{3}c_{H^{+}}\right)}^{\frac{2}{\beta_{1}+\alpha_{3}}}{\left(b_{1}k_{1}\right)}^{-\frac{2}{\beta_{1}+\alpha_{3}}}c_{\mathrm{Zn},0}{}^{-1}c_{{\mathrm{Co}}^{2+},0}$$

According to the experimental data of Lew [4] (Page 47, Figure 4-10), the logarithmic ratio ($ln(c_{{\mathrm{Co}}^{2+},0}c_{{\mathrm{Co}}^{2+},\mathrm{eq}}^{-1})$) of the equilibrium to the initial concentration of cobalt ions was approximately 4.2 at pH 3. It is assumed that $ln(c_{{\mathrm{Co}}^{2+},0}c_{{\mathrm{Co}}^{2+},\mathrm{eq}}^{-1})\in \left[3,8\right]$ considering the model simplification error. Accordingly, the following constraint can be imposed:

(35) $$2{\left(\beta_{1}+\alpha_{3}\right)}^{-1}\left(ln(k_{1}b_{1}k_{3}^{-1})+3ln(10)\right)-ln(b_{2})+ln(c_{\mathrm{Zn},0})\;\in \;\left[3,8\right]$$

Moreover, the forward rate of zinc dissolution Reaction (1) considered in the mixed-potential equation is assumed to be less than the reverse rate thereby leading to the following constraint:

(36) $$c_{{\mathrm{Zn}}^{2+}}exp(-\frac{\left(2-\beta_{1}\right)FE_{\mathrm{est}}}{RT})\;\leq \;\frac{1}{2}b_{1}\frac{c_{\mathrm{Zn}}}{c_{\mathrm{Zn},0}}exp(\frac{\beta_{1}FE_{\mathrm{est}}}{RT})$$

Mixed-potential estimation Equation (16) is substituted into the above equation while assuming $c_{\mathrm{Zn}}\approx c_{\mathrm{Zn},0}$. Meanwhile, given that the initial concentration of zinc ions (151 g/L) is far greater than the initial concentration of elementary zinc (4 g/L), it is further assumed that $c_{{\mathrm{Zn}}^{2+}}\approx c_{{\mathrm{Zn}}^{2+},0}$, i.e., the concentration of zinc ions remains constant. Accordingly, the following constraint can be imposed:

(37) $$2{\left(\beta_{1}+\alpha_{3}\right)}^{-1}\left(ln(b_{1}k_{1}k_{3}^{-1})+3ln(10)\right)-ln(b_{1})+ln(c_{{\mathrm{Zn}}^{2+},0})\;\leq \;-ln(2)$$

#### 2.3.2. Constraints on Hydrolysis Parameters

The hydrolysis Reaction (4) affects the rate of cobalt displacement reaction mainly by releasing hydrogen ions and consequently alleviating the decreasing trend of hydrogen ion concentration. Hereinafter, the simplified model is analyzed to derive an equation to estimate the attenuation coefficient for the apparent rate of hydron ion consumption in the presence of zinc ion hydrolysis. Next, the estimation equation is used to derive a constraint on hydrolysis parameter *b*_4_. The hydrolysis Reaction (4) generally has large forward and reverse rate constants and thereby can be treated as a quasi-equilibrium reaction namely *r*_4_ = 0. Therefore:

(38) $$c_{{\mathrm{Zn}}^{2+}}-b_{4}c_{\mathrm{Zn}{\left(\mathrm{OH}\right)}_{2}}c_{H^{+}}^{2}\;=\;0$$

Taking the total differential of the above equation:

(39) $$\delta c_{{\mathrm{Zn}}^{2+}}-b_{4}c_{H^{+}}^{2}\delta c_{\mathrm{Zn}{\left(\mathrm{OH}\right)}_{2}}-2b_{4}c_{\mathrm{Zn}{\left(\mathrm{OH}\right)}_{2}}c_{H^{+}}\delta c_{H^{+}}=0$$

Taking the total differential of state Equation (12) and material balance Equation (13):

(40) $$\left\{\begin{array}{l} \delta x_{i}\left(t\right)=r_{i}\delta t,i\;=\;1,2,3\\ \delta c_{{\mathrm{Zn}}^{2+}}\;=\;-\delta x_{1}\left(t\right)-\delta x_{4}\left(t\right)\\ \delta c_{H^{+}}\;=\;-2\delta x_{3}\left(t\right)+2\delta x_{4}\left(t\right)\\ \delta c_{\mathrm{Zn}{\left(\mathrm{OH}\right)}_{2}}\;=\;\delta x_{4}\left(t\right) \end{array}\right.$$

Substitution of Equation (40) into Equation (39) leads to:

(41) $$\frac{\delta x_{4}\left(t\right)}{\delta t}\;=\;\frac{4b_{4}c_{\mathrm{Zn}{\left(\mathrm{OH}\right)}_{2}}c_{H^{+}}}{\left(1+b_{4}c_{H^{+}}^{2}+4b_{4}c_{\mathrm{Zn}{\left(\mathrm{OH}\right)}_{2}}c_{H^{+}}\right)}r_{3}-\frac{1}{\left(1+b_{4}c_{H^{+}}^{2}+4b_{4}c_{\mathrm{Zn}{\left(\mathrm{OH}\right)}_{2}}c_{H^{+}}\right)}r_{1}$$

A combination of Equation (41) with Equation (23) of equilibrium mixed potential:

(42) $$\frac{\delta x_{4}\left(t\right)}{\delta t}\;=\;\frac{4b_{4}c_{\mathrm{Zn}{\left(\mathrm{OH}\right)}_{2}}c_{H^{+}}+1}{\left(1+b_{4}c_{H^{+}}^{2}+4b_{4}c_{\mathrm{Zn}{\left(\mathrm{OH}\right)}_{2}}c_{H^{+}}\right)}r_{3}+\frac{1}{\left(1+b_{4}c_{H^{+}}^{2}+4b_{4}c_{\mathrm{Zn}{\left(\mathrm{OH}\right)}_{2}}c_{H^{+}}\right)}r_{2}$$

According to Equations (40) and (42), the rate of change of hydrogen ion concentration with time is:

(43) $${\dot{c}}_{H^{+}}\;=\;\frac{\delta c_{H^{+}}}{\delta t}\;=\;\frac{-2\delta x_{3}\left(t\right)+2\delta x_{4}\left(t\right)}{\delta t}\;=\;-\frac{2b_{4}c_{H^{+}}^{2}}{\left(1+b_{4}c_{H^{+}}^{2}+4b_{4}c_{\mathrm{Zn}{\left(\mathrm{OH}\right)}_{2}}c_{H^{+}}\right)}r_{3}+\frac{2}{\left(1+b_{4}c_{H^{+}}^{2}+4b_{4}c_{\mathrm{Zn}{\left(\mathrm{OH}\right)}_{2}}c_{H^{+}}\right)}r_{2}$$

The second term in equation (43) incorporates the impact of cobalt removal rate $r_{2}$ on the rate of change of hydrogen ion concentration. Close examination of the experimental data of Lew [4] (Page 58, Figure 4-27) reveals that the pH evolution curve is only slightly affected by cobalt ion concentration thereby allowing one to ignore the second term of the above equation. In addition, rearrangement of hydrolysis equilibrium Equation (38) gives:

(44) $$c_{\mathrm{Zn}{\left(\mathrm{OH}\right)}_{2}}\;=\;b_{4}^{-1}c_{{\mathrm{Zn}}^{2+}}c_{H^{+}}^{-2}$$

Substitution of Equation (44) into Equation (43) leads to Equation (45):

(45) $${\dot{c}}_{H^{+}}\;=\;-2b_{4}c_{H^{+}}^{2}{\left(1+b_{4}c_{H^{+}}^{2}+4c_{{\mathrm{Zn}}^{2+}}c_{H^{+}}^{-1}\right)}^{-1}r_{3}$$

As shown above, zinc ion hydrolysis (4) alleviates the declining trend of hydrogen ion concentration. Therefore, the attenuation coefficient for the apparent consumption rate of hydrogen ions relative to the rate of hydrogen ion reduction (3) is less than one:

(46) $$\lambda_{H^{+}}\;=\;b_{4}c_{H^{+}}^{2}{\left(\left(1+b_{4}c_{H^{+}}^{2}+4c_{{\mathrm{Zn}}^{2+}}c_{H^{+}}^{-1}\right)\right)}^{-1}$$

The t-pH curve generated under the condition of zero initial concentration of zinc ions by Lew [4] (Page 70, Figure 4-42) showed that the rising slope of pH was much greater when the initial concentration of zinc ions was low than when it was high in the early stage (0–5 min) (Page 42, Figure 4-4). This trend suggests that the presence of zinc ions has a significant impact on the apparent rate of hydrogen evolution. Therefore, the attenuation coefficient $\lambda_{H^{+}}$ should be much less than 1 in the presence of zinc ions. Moreover, the third term of the denominator, which is a zinc ion-related term, is the largest one among the three terms: $4c_{{\mathrm{Zn}}^{2+}}c_{H^{+}}^{-1}$. Accordingly, the attenuation coefficient can be approximated as:

(47) $$\lambda_{H^{+}}\;\approx \;b_{4}c_{H^{+}}^{2}{\left(4c_{{\mathrm{Zn}}^{2+}}c_{H^{+}}^{-1}\right)}^{-1}$$

The experimental data of Lew [4] (Page 42, Figure 4-4) showed that when the pH was above 4.4, the slope of the pH curve was much smaller than that in the initial stage. It is assumed here that ${\left.\lambda_{H^{+}}\right|}_{\mathrm{pH}=4.4}\in \left[0.01,0.1\right]$ and $c_{{\mathrm{Zn}}^{2+}}\approx c_{{\mathrm{Zn}}^{2+},0}$. Taking the logarithm of $\lambda_{H^{+}}$, one has the following constraint on hydrolysis rate constant $b_{4}$:

(48) $$ln(b_{4}c_{{\mathrm{Zn}}^{2+},0}^{-1})\;\in \;\left[11.2\times ln(10\right)+ln(4),12.2\times ln(10)+ln(4)]$$

In this section, to reduce the searching space of parameters, the constraints on parameters are derived based on the approximation of experimental phenomenon metrics. However, the approximation formula of experimental phenomenon metrics can also be useful in parameter adjustment and optimizing. For example, Equation (28) for apparent first-order reaction rate of cobalt removal, can be used for determining the rough range of the required zinc loading and pH setting value under the constraints about cobalt removal rates. Equations (28), (31), (34), (47) also provide some intuitive information about the relation between the model parameters and process dynamical behavior, and thus would be useful for adjustment of model parameters while the dynamic changes in practical cobalt removal process.

#### 2.3.3. Constrained Parameter Estimation Problem

Introduction of constraints (Equations (26), (29), (32), (35), (37) and (48)) into the parameter estimation problem (22) leads to a constrained parameter estimation problem:

(49) $$\underset{p^{\left(1\right)}}{min}\overset{5}{\underset{j=1}{\sum }}\overset{n_{\mathrm{Co},j}}{\underset{i=1}{\sum }}\left(\frac{1}{n_{\mathrm{Co},j}}\left|ln(\frac{c_{{\mathrm{Co}}^{2+},0}^{\left(j\right)}}{{\hat{c}}_{{\mathrm{Co}}^{2+}}^{\left(j\right)}\left(t_{i,{\mathrm{Co}}^{2+}}^{\left(j\right)}\right)})-ln(\frac{c_{{\mathrm{Co}}^{2+},0}^{\left(j\right)}}{c_{{\mathrm{Co}}^{2+},i}^{\left(j\right)}})\right|\right)+\frac{\lambda_{\mathrm{pH}}}{n_{\mathrm{pH}}}\overset{n_{\mathrm{PH}}}{\underset{l=1}{\sum }}\left|-{log}_{10}({\hat{c}}_{H^{+}}^{\left(1\right)}\left(t_{l,H^{+}}^{\left(1\right)}\right))-{\mathrm{pH}}_{l}\right|\;s.t.\left[\begin{array}{c} {\hat{c}}_{{\mathrm{Co}}^{2+}}^{\left(j\right)}\left(t\right)\\ {\hat{c}}_{H^{+}}^{\left(j\right)}\left(t\right) \end{array}\right]\;=\;\left\{\begin{array}{c} G(t|p^{\left(1\right)},c_{0}^{\left(j\right)}),\mathrm{if}\;\;j=1,\\ G^{*}(t|p^{\left(1\right)},c_{0}^{\left(j\right)}),\mathrm{if}\;j>1, \end{array}\right.\;\beta_{1}\in \left[0.7,1.5\right],\alpha_{2}\;\in \;\left[0.2,0.8\right],\alpha_{3}\;\in \;\left[0.2,0.8\right],\;\left(26\right),\left(29\right),\left(32\right),\left(35\right),\left(37\right),\left(48\right).$$

Equation (49) contains nonlinear constraints (Equations (26), (29), (32), (35), (37) and (48)). Gradient-based constrained optimization algorithms such as the sequential quadratic programming algorithm and the interior-point algorithm can only handle convex constrained optimization or quasi-convex constrained optimization problems while Equation (49) is a non-convex constrained optimization problem and cannot be solved using such algorithms. Heuristic optimization algorithms are effective in solving non-convex, box-constrained optimization problems; i.e., each decision variable is bounded by two constant values as the upper and lower limits. However, because nonlinear constraints generally come in different forms and are difficult to handle in a unified manner, parameter optimization would be far less satisfactory under nonlinear constraints than under box constraints. A more serious problem is that most intelligent constrained optimization methods require sampling outside the feasible region such as various heuristic optimization algorithms with penalty functions.

In Equation (49), however, most of the parameters outside the feasible region are rigid parameters, which will cause the state-space model to be unsolvable and thus seriously affect the optimization efficiency. Therefore, the constraints contained in Equation (49) will be analyzed in the next sub-section to transform Equation (49) into an easy-to-solve box-constrained optimization problem so that a general heuristic optimization algorithm can be used to iteratively optimize the parameters within the feasible region of the constraints to achieve higher solution efficiency and accuracy.

### 2.4. Parameter Transformation before Estimation

Constraints (Equations (26), (29), (32), (35), (37) and (48)) will be linear constraints in which are linearly combined if are ignored. Optimization problems with linear constraints can be transformed to a box-constrained optimization problem through linear mapping of the parameters to be estimated. Let:

(50) $$p^{\left(1\right)}\;=\;{\left[,ln(k_{1}\right),ln(k_{2}),ln(k_{3}),ln(b_{1}),ln(b_{2}),ln(b_{4})]}^{T}$$

It is obvious that the following equation holds:

(51) $$p^{\left(1\right)}\;=\;\left[\beta_{1},\alpha_{2},\alpha_{3},{\left(P^{\left(1,2\right)}\right)}^{T}\right]$$

Equation (49) is now re-formulated as:

(52) $$\underset{\beta_{1},\alpha_{2},\alpha_{3},p^{\left(1,2\right)}}{min}\overset{5}{\underset{j=1}{\sum }}\overset{n_{\mathrm{Co},j}}{\underset{i=1}{\sum }}\left(\frac{1}{n_{\mathrm{Co},j}}\left|ln(\frac{c_{{\mathrm{Co}}^{2+},0}^{\left(j\right)}}{{\hat{c}}_{{\mathrm{Co}}^{2+}}^{\left(j\right)}\left(t_{i,{\mathrm{Co}}^{2+}}^{\left(j\right)}\right)})-ln(\frac{c_{{\mathrm{Co}}^{2+},0}^{\left(j\right)}}{c_{{\mathrm{Co}}^{2+},i}^{\left(j\right)}})\right|\right)+\frac{\lambda_{\mathrm{pH}}}{n_{\mathrm{pH}}}\overset{n_{\mathrm{PH}}}{\underset{l=1}{\sum }}\left|-{log}_{10}({\hat{c}}_{H^{+}}^{\left(1\right)}\left(t_{l,H^{+}}^{\left(1\right)}\right))-{\mathrm{pH}}_{l}\right|\;s.t.\left[\begin{array}{c} {\hat{c}}_{{\mathrm{Co}}^{2+}}^{\left(j\right)}\left(t\right)\\ {\hat{c}}_{H^{+}}^{\left(j\right)}\left(t\right) \end{array}\right]\;=\;\left\{\begin{array}{c} G(t|p^{\left(1\right)},c_{0}^{\left(j\right)}),\mathrm{if}\;\;j=1,\\ G^{*}(t|p^{\left(1\right)},c_{0}^{\left(j\right)}),\mathrm{if}\;\;j>1, \end{array}\right.\;\beta_{1}\;\in \;\left[0.7,1.5\right],\alpha_{2}\in \left[0.2,0.8\right],\alpha_{3}\;\in \;\left[0.2,0.8\right],\;A\left(\beta_{1},\alpha_{2},\alpha_{3}\right)p^{\left(1,2\right)}+B\left(\beta_{1},\alpha_{2},\alpha_{3}\right)\;\in \;\left[z_{min},z_{max}\right]$$

Where

(53) $$A\left(\beta_{1},\alpha_{2},\alpha_{3}\right)\;=\;\left[\begin{array}{cccccc} -{\left(\beta_{1}+\alpha_{3}\right)}^{-1} & 0 & {\left(\beta_{1}+\alpha_{3}\right)}^{-1} & -{\left(\beta_{1}+\alpha_{3}\right)}^{-1} & 0 & 0\\ \alpha_{2}{\left(\beta_{1}+\alpha_{3}\right)}^{-1} & 1 & -\alpha_{2}{\left(\beta_{1}+\alpha_{3}\right)}^{-1} & \alpha_{2}{\left(\beta_{1}+\alpha_{3}\right)}^{-1} & 0 & 0\\ \left(\alpha_{3}-\alpha_{2}\right){\left(\beta_{1}+\alpha_{3}\right)}^{-1} & -1 & \left(\beta_{1}+\alpha_{2}\right){\left(\beta_{1}+\alpha_{3}\right)}^{-1} & \left(\alpha_{3}-\alpha_{2}\right){\left(\beta_{1}+\alpha_{3}\right)}^{-1} & 0 & 0\\ 2{\left(\beta_{1}+\alpha_{3}\right)}^{-1} & 0 & -2{\left(\beta_{1}+\alpha_{3}\right)}^{-1} & 2{\left(\beta_{1}+\alpha_{3}\right)}^{-1} & -1 & 0\\ 2{\left(\beta_{1}+\alpha_{3}\right)}^{-1} & 0 & -2{\left(\beta_{1}+\alpha_{3}\right)}^{-1} & 2{\left(\beta_{1}+\alpha_{3}\right)}^{-1}-1 & 0 & 0\\ 0 & 0 & 0 & 0 & 0 & 1 \end{array}\right]$$

(54) $$B\left(\beta_{1},\alpha_{2},\alpha_{3}\right)\;=\;\left[\begin{array}{l} -\frac{ln(10^{3})}{\beta_{1}+\alpha_{3}}\\ \frac{ln(10^{3})\alpha_{2}}{\beta_{1}+\alpha_{3}}+ln(c_{\mathrm{Zn},0})\\ \frac{ln(10^{3})\left(\alpha_{3}-\alpha_{2}\right)}{\beta_{1}+\alpha_{3}}\\ \frac{ln(10^{6})}{\beta_{1}+\alpha_{3}}+ln(c_{\mathrm{Zn},0})\\ \frac{ln(10^{6})}{\beta_{1}+\alpha_{3}}+ln(c_{{\mathrm{Zn}}^{2+},0})\\ -ln(c_{{\mathrm{Zn}}^{2+},0}) \end{array}\right]$$

(55) $$z_{min}\;=\;{\left[-0.7\frac{F}{RT}\;,\ln\left(0.015\right),\ln\left(5\right),3,-\infty ,11.2\times \ln\left(10\right)+\ln\left(4\right)\right]}^{T}$$

(56) $$z_{max}\;=\;{\left[-0.5\;\frac{F}{RT}\;,\;ln\left(0.060\right),ln\left(10\right),8,-ln\left(2\right),,12.2\times ln\left(10\right)+ln\left(4\right)\right]}^{T}$$

The parameters to be estimated in Equation (52) are transformed through quasi-linear mapping:

(57) $$z\;=\;A\left(\beta_{1},\alpha_{2},\alpha_{3}\right)p^{\left(1,2\right)}+B\left(\beta_{1},\alpha_{2},\alpha_{3}\right)$$

As indicated by the structure of $A\left(\beta_{1},\alpha_{2},\alpha_{3}\right)$, this matrix is an invertible matrix. Accordingly:

(58) $$p^{\left(1,2\right)}\;=\;A{\left(\beta_{1},\alpha_{2},\alpha_{3}\right)}^{-1}\left(z-B\left(\beta_{1},\alpha_{2},\alpha_{3}\right)\right)$$

Equation (52) now can be transformed to problem Equation (59) as follows:

(59) $$\underset{\beta_{1},\alpha_{2},\alpha_{3},z}{min}\overset{5}{\underset{j=1}{\sum }}\overset{n_{\mathrm{Co},j}}{\underset{i=1}{\sum }}\left(\frac{1}{n_{\mathrm{Co},j}}\left|ln(\frac{c_{{\mathrm{Co}}^{2+},0}^{\left(j\right)}}{{\hat{c}}_{{\mathrm{Co}}^{2+}}^{\left(j\right)}\left(t_{i,{\mathrm{Co}}^{2+}}^{\left(j\right)}\right)})-ln(\frac{c_{{\mathrm{Co}}^{2+},0}^{\left(j\right)}}{c_{{\mathrm{Co}}^{2+},i}^{\left(j\right)}})\right|\right)+\frac{\lambda_{\mathrm{pH}}}{n_{\mathrm{pH}}}\overset{n_{\mathrm{PH}}}{\underset{l=1}{\sum }}\left|-{log}_{10}({\hat{c}}_{H^{+}}^{\left(1\right)}\left(t_{l,H^{+}}^{\left(1\right)}\right))-{\mathrm{pH}}_{l}\right|\;s.t.\left[\begin{array}{c} {\hat{c}}_{{\mathrm{Co}}^{2+}}^{\left(j\right)}\left(t\right)\\ {\hat{c}}_{H^{+}}^{\left(j\right)}\left(t\right) \end{array}\right]\;=\;\left\{\begin{array}{c} G(t|A\left(\beta_{1},\alpha_{2},\alpha_{3}{)}^{-1}\left(z-B\left(\beta_{1},\alpha_{2},\alpha_{3}\right)\right),c_{0}^{\left(j\right)}\right),\mathrm{if}\;j=1,\\ G^{*}(t|A\left(\beta_{1},\alpha_{2},\alpha_{3}{)}^{-1}\left(z-B\left(\beta_{1},\alpha_{2},\alpha_{3}\right)\right),c_{0}^{\left(j\right)}\right),\mathrm{if}\;j\rangle 1, \end{array}\right.\;\beta_{1}\in \left[0.7,1.5\right],\alpha_{2}\;\in \;\left[0.2,0.8\right],\alpha_{3}\in \left[0.2,0.8],z\in [z_{min},z_{max}\right]$$

Equation (59) is a pure box-constrained optimization problem and such a problem can be solved using the general PSO algorithm [14]. Meantime, as indicated by the constraint construction process in Section 2.3.1 and Section 2.3.2, some parameters of z are actually linearly related to some experimental metrics (e.g., mixed potential, apparent first-order reaction rate constant of cobalt, equilibrium concentration); $z_{min}$ and $z_{max}$ are the transformed lower and upper limits of the experimental metrics, respectively. Therefore, the proper solution of Equation (59) is an estimation of value ranges for experimentally measurable metrics that are transformed from kinetic model parameters whose value ranges are difficult to estimate directly.

In summary, the proposed parameter estimation algorithm for cobalt–hydron electrochemical competition is as follows (Algorithm 1):
**Algorithm 1:** Constrained parameter estimation algorithm (CPEA) The inputs include the following: monitored cobalt ion concentration, monitored pH $c_{{\mathrm{Co}}^{2+},i}^{\left(j\right)},j=1,\ldots ,5,i=1,\ldots ,n_{\mathrm{Co},j},\;{\mathrm{pH}}_{l},l=1,\ldots ,n_{\mathrm{pH}}$ initial concentration vector
$c_{0}^{\left(j\right)}$
initial mixed potential
$E_{0}$
weight coefficient
$\lambda_{\mathrm{pH}}$ Npop (the population size of the PSO algorithm), and MaxIters (the maximum number of iterations).

Output: Cobalt–hydrogen electrochemical competition model parameters
$p^{\left(1\right)}$
 Steps:
(1)Use the PSO algorithm to solve Equation (59) for $\beta_{1},\alpha_{2},\alpha_{3},z$(2)Calculate $p^{\left(1,2\right)}$ according to Equation (58)(3)Calculate $p^{\left(1\right)}$ according to Equation (51)

## 3. Model and Algorithm Verification

The solution of Equation (59) requires one to know the initial concentration of the reactants and the initial mixed potential in each set. The initial concentration vector of test (*j*) is as follows:

(60) $$c_{0}^{\left(j\right)}\;=\;{\left[2.310,6.12\times 10^{-2},0.44\times 10^{-3},0,10^{-pH_{0,j}},2.310b_{4}^{-1}{\left(c_{H^{+},0}^{\left(j\right)}\right)}^{-2}\right]}^{T}\mathrm{mol}/L$$

where *pH*_0_ is the initial pH vector:

(61) $$pH_{0}\;=\;{\left[3.7\;\;3.0\;\;3.6\;\;4.0\;\;4.4\right]}^{T}$$

The fifth term of Equation (60) represents the initial Zn(OH)_2_ concentration, which can be calculated from the equilibrium Equation (38): $c_{\mathrm{Zn}{\left(\mathrm{OH}\right)}_{2},0}=c_{{\mathrm{Zn}}^{2+},0}b_{4}^{-1}c_{H^{+},0}^{-2}$. The initial mixed potential can be estimated by solving the equilibrium mixed-potential Equation (23) as follows:

(62) $$E_{0}\;=\;\underset{E}{arg}\left(\overset{3}{\underset{i=1}{\sum }}2r_{i}\left(c_{0},E\right)=0\right)$$

The weight coefficient $\lambda_{\mathrm{pH}}$ is set to 50. The PSO algorithm is used to optimally solve Equation (59) with Npop set to 50 and MaxIters set to 50. The state-space model (13) is solved using the stiff differential equation solver ode15s in Matlab 2016a. During the optimization process, some randomly generated parameters may make the state-space model very stiff. To avoid wasting too much time on the solution of strongly stiff problems, the time step of the ode15s solver is set to $1\times 10^{-8}$.

Table 1 lists the model parameters optimized by the constrained parameter estimation algorithm. Meanwhile, to evaluate the impact of constraints on the solution, the original parameter estimation problem PRO-1 without constraints is also solved by using the PSO algorithm directly but under two setting conditions—namely Npop = 50 and MaxIters = 50 versus Npop = 500 and MaxIters = 50. The PSO algorithm requires a pre-specified range of parameter values, but Equation (22) only specifies value ranges for $\beta_{1},\alpha_{2},\mathrm{and}\;\alpha_{3}$. Therefore, the search range of $p^{\left(1,2\right)}$ is specified for the original parameter estimation problem using the results of the constrained parameter estimation:

(63) $$p^{\left(1,2\right)}\;\in \;\left[p_{\mathrm{best}}^{\left(1,2\right)}-10,p_{\mathrm{best}}^{\left(1,2\right)}+10\right]$$

Here, $p_{\mathrm{best}}^{\left(1,2\right)}$ is the result of constrained parameter estimation with its component values listed in Table 1.

**Figure 3:**
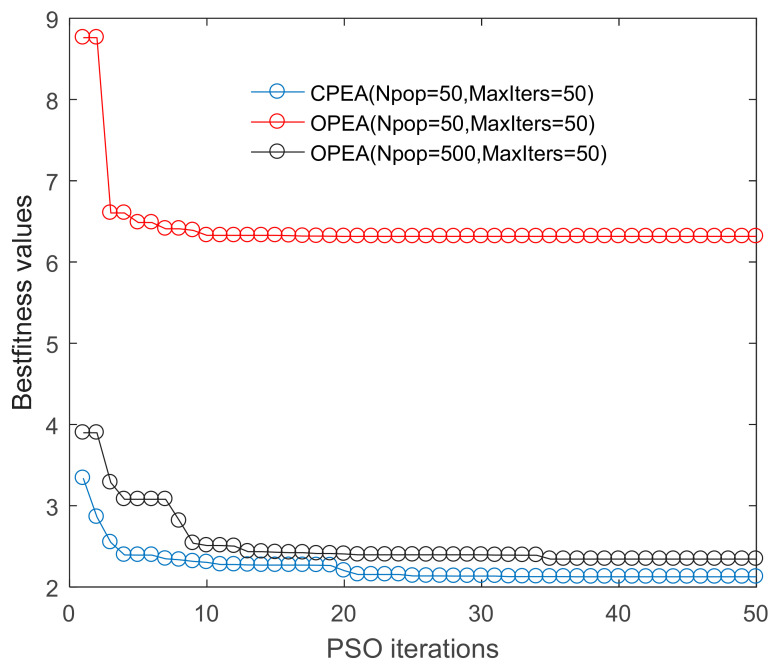
The iteration curves of identification algorithms. CPEA: constrained parameter estimation algorithm, OPEA: original parameter estimation algorithm.

The curves of the best fitness values versus the number of iterations are compared between the proposed constrained parameter estimation algorithm and the original parameter estimation algorithm(OPEA) (Figure 3), and the two algorithms are also compared in terms of estimation accuracy and optimization time (Table 2). Table 2 shows that the original parameter estimation algorithm achieved a relative fitting error of 74.1% for the cobalt concentration curve under the setting condition of Npop = 50 and MaxIters = 50; this failed to find a meaningful solution. Only when the population size Npop increased to 500 could a meaningful solution be found, but the spent time substantially increased by nearly a factor of 8 versus the constrained parameter estimation algorithm. Meanwhile, the mean relative fitting error for the logarithmic concentration of cobalt ions was also larger for the conventional PSO algorithm. These observations indicated that the constrained parameter estimation algorithm significantly improved the estimation efficiency. Moreover, as shown by the curves in Figure 3, the original PSO algorithm with Npop = 50 failed to converge to a good solution. Only when the population size was increased to 500 could the conventional PSO algorithm achieve a parameter estimation accuracy close to that of the constrained PSO algorithm. However, the former algorithm still gave a slightly higher best fitness value than the latter algorithm indicating that constrained parameter estimation is more accurate. It was also reflected on the mean relative errors for fitting the curves of cobalt ion concentrations (Table 2): the MSE from the CPEA, 7.142%, was lower than that of the OPEA,8.775%.

**Figure 4:**
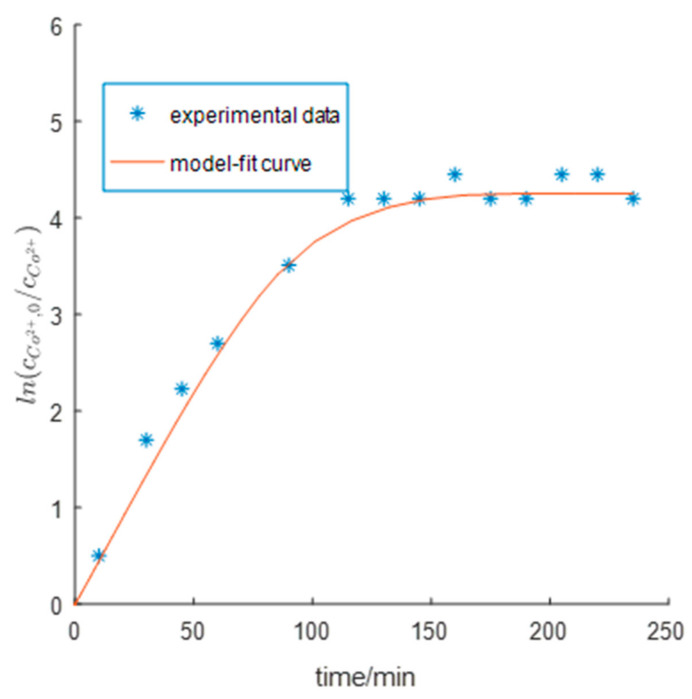
Comparing of the model-fit and experimental [14] dynamical cobalt concentration curves (experimental data come from Lew [4], Page 47, Figure 4-10) with uncontrolled pH started from 3.7; experimental time: 250 min.

**Figure 5:**
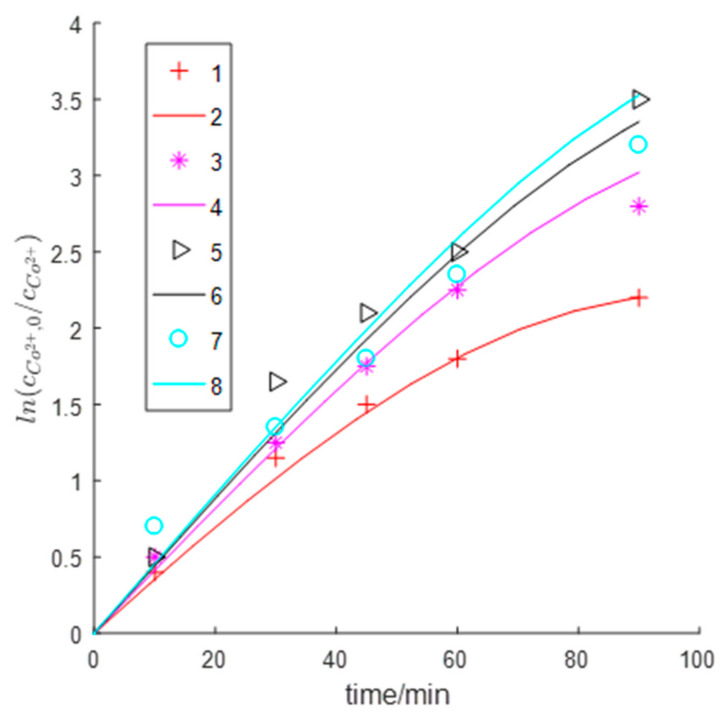
Comparing model-fit and experimental dynamical cobalt concentration curves (experimental data from Lew [4], Page 62, Figure 4-31). The pH is controlled to be a constant value. Symbols 1, 3, 5, and 7 represent experimental data at pH 3, pH 3.6, pH 4, and pH 4.4, respectively; lines 2, 4, 6, and 8 represent fitted lines at pH 3, pH 4, pH 4, and pH 4.4, respectively.

The simulation was performed based on the results of constrained parameter estimation. Model-fit values versus experimentally measured values of cobalt ion concentration were plotted in Figure 4 and Figure 5. The model accurately fitted the curves of cobalt ion concentration versus time under different pH conditions with a mean relative fitting error of 7.142%. The cobalt displacement rate was the lowest at pH 3. The model accurately predicted the inhibitory effect of the competitive hydrogen evolution on cobalt removal when the pH was too high. The model was accurate in predicting the cobalt removal rate at pH 3 and pH 3.6. However, the model had a large error in predicting the cobalt removal rate at pH 4.4, which may be related to the “passivation” effect in the actual cobalt removal process. In other words, Zn(OH)_2_ is formed and covers the zinc dust surface at high pH, which causes the zinc dust to partially lose reactivity thereby decreasing the cobalt removal rate. The proposed model failed to consider this process and thereby overestimated the cobalt removal rate. The cobalt displacement Reaction (2) gradually reached equilibrium with time as the decrease in cobalt ion concentration gradually leveled off (Figure 4); this trend was well fit by the model.

**Figure 6:**
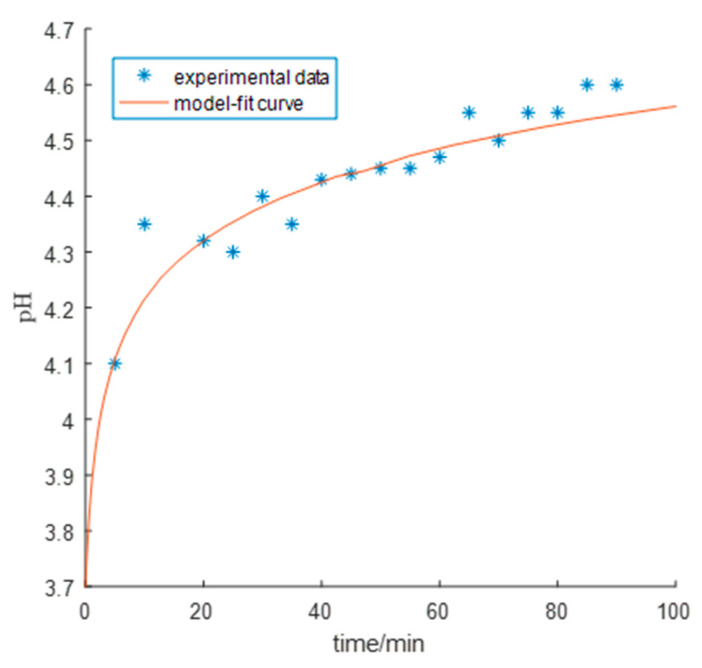
Comparing the model-fit and experimental [14] dynamical pH curves (experimental data come from Lew [4], Page 42, Figure 4-4) with uncontrolled pH started from 3.7.

The curve of monitored pH versus time was well fitted by the proposed model (Figure 6) with a mean relative fitting error of 0.729% and a goodness-of-fit of 0.86. In the initial stage, the pH rapidly increased due to massive hydrogen evolution. The addition of zinc dust promoted the forward reaction of zinc hydrolysis (4), which replenished hydrogen ions. As a result, the apparent consumption rate of hydrogen ions was far less than the actual hydrogen evolution rate, which led to a dramatic decrease in the rate of change of pH with time thereby leading to a monotonic and concave-down curve.

## 4. Conclusions

(1) For the first time, a mechanistic kinetic model of cobalt–hydrogen electrochemical competition was proposed for the cobalt removal process in zinc hydrometallurgy by exploring the mechanism of the impact of hydrogen ions on the cobalt removal rate and the mechanism of zinc ion hydrolysis. The model parameters were estimated based on experimental data. The estimation results showed that the model accurately simulated the competitive inhibitory effect of hydrogen ions on cobalt removal and the trend of pH in the cobalt removal process. It shows a goodness-of-fit value of 0.982 on cobalt concentration values and that of 0.860 on pH values.
(2) The difficulty in using heuristic optimization algorithms to estimate the parameters of reaction kinetic models with little information about the parameter value ranges was addressed. A new approach for estimating the parameters of strongly nonlinear and chemical reaction mechanism models was proposed. Specifically, parameter estimation constraints were first constructed based on an estimation of the value ranges of experimental metrics to narrow the search space. Next, the nonlinear constraints were transformed to simple box constraints through quasi-linear mapping, and a box-constrained parameter estimation scheme was constructed for the cobalt–hydrogen electrochemical competition model. Parameter estimation tests showed that the constrained parameter estimation algorithm was far more efficient than the conventional parameter estimation algorithm. Specifically, the constrained parameter estimation algorithm reduced the solution time by 90 percent. Furthermore, the proposed algorithm had a lower MSE on fitting the cobalt ion concentration curves than the conventional parameter estimation algorithm (7.142% vs. 8.775%).
(3) The non-linearity of the electrochemical and hydrolysis reactions involved in the cobalt removal process as well as the high coupling between multiple reactions made the model incomprehensible. To overcome this limitation, we derived estimation equations for the value ranges of relevant experimental metrics—mixed potential, apparent first-order reaction rate of cobalt removal, equilibrium concentration, and attenuation coefficient for the apparent consumption rate of hydrogen ions—based on model simplification assumptions in the constraint construction process. These equations linked the experimental phenomenon metrics to the model parameters providing deeper insights into the model parameters adjustment for model users and controller design [22,23].
(4) As stated above, the model proposed in this paper can accurately predict the change of cobalt ion concentration and the rate of hydrogen replacement reaction during the cobalt removal process, so it is helpful for optimizing the zinc powder and acid usage in the practical cobalt removal process of zinc hydrometallurgy, although this may require for adding extra equations about the relationship between dosages and the zinc powder concentration or pH. In addition, the identification framework proposed significantly reduces the time required for parameter identification of the model, which is also very helpful for improving the efficiency and accuracy of the modelling in the optimal control implementation.
(5) The model does not consider the “passivation” effect of zinc ion, as a result, it has a large error in predicting the cobalt removal rate while the value of controlled pH is larger than 4.4. Therefore, the available pH value of the model should be lower than 4.4. In addition, the model does not consider the catalytic effect of copper and antimony, so it is not suitable for the process with large fluctuations in copper and antimony concentrations.

## Figures and Tables

**Table 1:** Results of constraints guide identification.

Parameter	Value	Parameter	Value	Parameter	Value
$\beta_{1}$	1.004	$ln\left(k_{1}\right)$	−11.00	$ln(b_{1})$	38.09
$\alpha_{2}$	0.299	$ln(k_{2})$	−8.298	$ln(b_{2})$	30.03
$\alpha_{3}$	0.603	$ln(k_{3})$	−13.87	$ln(b_{4})$	27.53

**Table 2:** Performances of parameter-identification algorithms.

No.	$ln(\frac{c_{Co^{2+},0}}{c_{Co^{2+}}})$		pH		Ts (min)
	MSE	Rsq	MSE	Rsq	
1	7.142%	0.982	0.729%	0.860	6
2	74.1%	−0.11	0.732%	0.859	7
3	8.775%	0.975	0.721%	0.863	56

1. Constrained parameter estimation algorithm (CPEA) with Npop = 50 and MaxIter s = 50; 2. Original parameter estimation algorithm (OPEA) with Npop = 50 and MaxIters = 50; 3. Original parameter estimation algorithm (OPEA) with Npop = 500 and MaxIters = 50; MSE: mean relative error; Rsq: goodness-of-fit; Ts, solution time.

## Data Availability

Publicly available datasets were analyzed in this study, reference number [4]. It can be found here: https://open.library.ubc.ca/cIRcle/collections/ubctheses/831/items/1.0078522.
